# Neuroprotection of Indole-Derivative Compound NC001-8 by the Regulation of the NRF2 Pathway in Parkinson's Disease Cell Models

**DOI:** 10.1155/2019/5074367

**Published:** 2019-10-31

**Authors:** Pei-Cih Wei, Guey-Jen Lee-Chen, Chiung-Mei Chen, Yih-Ru Wu, Yi-Jing Chen, Jia-Li Lin, Yen-Shi Lo, Ching-Fa Yao, Kuo-Hsuan Chang

**Affiliations:** ^1^Department of Neurology, Chang Gung Memorial Hospital-Linkou Medical Center, Chang Gung University School of Medicine, Taoyuan, Taiwan; ^2^Department of Life Science, National Taiwan Normal University, Taipei, Taiwan; ^3^Department of Chemistry, National Taiwan Normal University, Taipei, Taiwan

## Abstract

Parkinson's disease (PD) is a common neurodegenerative disease accompanied by a loss of dopaminergic (DAergic) neurons. The development of therapies to prevent disease progression is the main goal of drug discovery. There is increasing evidence that oxidative stress and antioxidants may contribute to the pathogenesis and treatment of PD, respectively. In the present study, we investigated the antioxidative protective effects of the indole-derivative compound NC001-8 in DAergic neurons derived from SH-SY5Y cells and PD-specific induced pluripotent stem cells (PD-iPSCs) carrying a *PARKIN* ex5del mutation. In SH-SY5Y-differentiated DAergic neurons under 1-methyl-4-phenylpyridinium (MPP^+^) treatment, NC001-8 remarkably reduced the levels of reactive oxygen species (ROS) and cleaved caspase 3; upregulated nuclear factor erythroid 2-related factor 2 (NRF2) and NAD(P)H dehydrogenase, quinone 1 (NQO1); and promoted neuronal viability. In contrast, *NRF2* knockdown abolished the effect of NC001-8 on the reduction of ROS and improvement of neuronal viability. In H_2_O_2_-treated DAergic neurons differentiated from PD-iPSCs, NC001-8 rescued the aberrant increase in ROS and cleaved caspase 3 by upregulating NRF2 and NQO1. Our results demonstrated the protective effect of NC001-8 in DAergic neurons via promoting the NRF2 antioxidative pathway and reducing ROS levels. We anticipate that our present *in vitro* assays may be a starting point for more sophisticated *in vivo* models or clinical trials that evaluate the potential of NC001-8 as a disease modifier for PD.

## 1. Introduction

Parkinson's disease (PD), the most common movement disorder worldwide, is characterized by rigidity, resting tremor, bradykinesia, and postural instability [1]. Loss of dopaminergic (DAergic) neurons in the substantia nigra pars compacta and formation of Lewy bodies with *α*-synuclein aggregation are the main pathological features of PD [2]. The disease is caused by several environmental factors and genetic mutations, which are still poorly understood and widely researched [3]. Genetic mutations of *ATP13A2*, *DJ1*, *HTRA2*, *LRRK2*, *PARKIN*, *PINK1*, *SNCA*, and *UCHL1* have been recognized as key mediators for early-onset PD [4]. These genes have been associated with the dysregulation of antioxidative stress, cell survival, apoptosis, mitochondrial function, and the ubiquitin-proteasome degradation system [4–7]. Among these potential pathogenic mechanisms, the increased generation of reactive oxygen species (ROS) has been previously identified in patients with PD and in PD models [7]. ROS enhance the translocation of the nuclear factor erythroid 2-related factor 2 (NRF2) into the nucleus. The activated NRF2 is associated with small musculoaponeurotic fibrosarcomas (*sMAF*) for binding to antioxidant response element (ARE). The interaction of NRF2 and ARE regulates downstream antioxidant-related genes, including NAD(P)H dehydrogenase, quinone 1 (*NQO1*), glutamate-cysteine ligase catalytic subunit (*GCLC*), hemeoxygenase-1 (*HMOX1*), and glutathione-S-transferase (*GST*) [8–10]. Furthermore, the NRF2 pathway has been demonstrated to promote neuronal cell survival in H_2_O_2_-treated SH-SY5Y cells and 1-methyl-4-phenyl-1,2,3,6-tetrahydropyridine- (MPTP)- treated mouse models of PD [11, 12]. Therefore, enhancement of the NRF2 antioxidative pathway has the potential to be a therapeutic strategy for PD [13–16].

Indole and its derivative compounds with an aromatic heterocyclic structure have been recently used in studies on neurodegenerative diseases [17–23]. Endogenous melatonin-related indole-3-propionic acid exhibited an effect against ROS damage and cell death resulting from the addition of A*β* peptide on SK-N-SH human neuroblastoma cells and primary rat hippocampal neurons [20]. Another endogenous indole derivative, indolepropionamide, was also shown to prolong rotifer lifespan and recover the mitochondrial metabolic function in rodents by decreasing the generation of free radicals [21]. We have previously demonstrated that the indole derivative NC001-8 is able to mitigate oxidative stress and polyglutamine (polyQ) aggregation by upregulated chaperones and/or autophagy in spinocerebellar ataxia-type (SCA) cell models [22, 23]. In the present study, we examined the effect of NC001-8 in MPP^**+**^-treated DAergic neurons derived from SH-SY5Y cells, focusing particularly on the maintenance of neuronal survival and reduction of ROS by increasing NRF2 and NQO1 expression. Furthermore, NC001-8 neuroprotective effects were also explored in DAergic neurons derived from induced pluripotent stem cells (iPSCs) carrying a *PARKIN* ex5del mutation (PD-iPSC) [24]. Our findings demonstrated that NC001-8 reduced oxidative stress via the enhancement of the NRF2 antioxidative pathway, suggesting the potential of NC001-8 in treating PD.

## 2. Materials and Methods

### 2.1. Culture and DAergic Differentiation of SH-SY5Y Cells

Human SH-SY5Y neuroblastoma cells (ATCC CRL-2266) were cultured in Dulbecco's Modified Eagle Medium/Nutrient Mixture F-12 (DMEM/F12) supplemented with 10% fetal bovine serum (FBS) (Invitrogen), 1x nonessential amino acid (NEAA, Invitrogen), and 1 mM sodium pyruvate (Invitrogen) at 37°C with 5% CO_2_. The differentiation of DAergic neurons was induced by incubation with 120 nM of TPA (Sigma-Aldrich) for 14 days. Cells were treated with MPP^+^ (1 mM, Sigma-Aldrich) and/or indole derivative NC001-8 (0.1 *μ*M) [22, 23] for 2 days and/or 14 days, respectively.

### 2.2. Maintenance of Human iPSCs and DAergic Neuronal Induction

The source of human iPSCs including NC1 and NC2 (normal control) and PD1 and PD2 (Parkinson's disease) was from our lab, and the maintenance of undifferentiated cells and the DAergic neuron induction were based on the previous protocol [24]. Briefly, human iPSCs (more than 50 passages) were cultured on mouse embryonic fibroblast feeder layers in Knockout DMEM (Invitrogen) supplemented with 20% Knockout Serum Replacement (Invitrogen), 8 ng/ml basic fibroblast growth factor (bFGF, Gibco), 50 U/ml penicillin (Invitrogen), 50 mg/ml streptomycin (Invitrogen), 1 mM sodium pyruvate, 1x NEAA, 2 mM L-glutamine (Invitrogen), and 0.1 mM 2-mercaptoethanol (Invitrogen). Differentiation of DAergic neurons was modified from the dual-inhibition monolayer differential described by Chambers et al. [25]. Initially, the inducing conditions were coupled with the N2B27 medium (Invitrogen), 10 *μ*M SB431542 (STEMCELL Technologies), and 500 ng/ml Noggin (STEMCELL Technologies). Neuronal growth was promoted by the addition of 20 ng/ml brain-derived neurotrophic factor (BDNF, PeproTech), 0.2 mM ascorbic acid (STEMCELL Technologies), 200 ng/ml Sonic hedgehog (STEMCELL Technologies), and 100 ng/ml FGF8 (Thermo Fisher Scientific) on days 9-12. Subsequently, matured neurons were obtained following 14 days of culture in the medium supplemented with 20 ng/ml BDNF (PeproTech), 0.2 mM ascorbic acid (STEMCELL Technologies), 10–20 ng/ml glial cell-derived neurotrophic factor (GDNF, PeproTech), and 1 ng/ml transforming growth factor *β*3 (TGF*β*3, PeproTech). Matured DAergic neurons were pretreated with 0.1 *μ*M NC001-8 for 6 days. A concentration of 100 *μ*M H_2_O_2_ was used for 8 hours to increase the ROS level in iPSC-derived DAergic neurons.

### 2.3. Extraction of RNA and Profiling of Relevant ROS Genes by the q-PCR Array

Total RNA was isolated using the Trizol reagent (Invitrogen). Reverse transcription (RT) was performed by superscript III (Invitrogen) with an initial concentration of 5 *μ*g total RNA. We established an in-house human panel for ROS profiling analysis with real-time qPCR using SYBR green reagents (Applied Biosystems). The gene entities of the q-PCR array are listed in the supplementary table ([Supplementary-material supplementary-material-1]). The thermocyclic conditions were as follows: 50°C for 2 min, 95°C for 10 min, 95°C for 15 s, and 60°C for 1 min for 40 cycles, which were through the ABI 7900 HT RT-PCR system (Applied Biosysple was assessed in triplicate. Relative expression values were normalized to *β*-*actin*. Relative gene expressions were calculated using the 2^-*ΔΔ*CT^ method, ΔC_T_ = C_T_ (target gene)–C_T_ (*β* − *actin*), in which C_T_ indicates cycle threshold (the fractional cycle number where the fluorescent signal reaches detection threshold). Primer sequences of 4 validated genes and the endogenous control are as follows: *NRF2* “CATGCCCTCACCTGCTACTT (forward)” and “TGTTCTGGTGATGCCACACT (reverse),” *NQO1* “TTACTATGGGATGGGGTCCA (forward)” and “TTTCAATGCACCACAAGAGG (reverse),” *SOD2* “GATGTGCATCAAGCCTGGTA (forward)” and “TGCAGACCTCTTTGATGGTTG (reverse),” *GCLM* “TCCTACCTGCACCCTCAACT (forward)” and “TGTGAACATCAGCCTGGAAA (reverse),” and *β-actin* “TCCCTGGAGAAGAGCTACGA (forward)” and “AGCACTGTGTTGGCGTACAG (reverse).”

### 2.4. Small Interfering RNA Transfection

The SH-SY5Y cells were transfected with siRNA for *NRF2* (Sigma-Aldrich) using the Lipofectamine® RNAiMAX Transfection Reagent (Invitrogen) according to the manufacturer's instructions. Furthermore, the expression of *NRF2* was confirmed by qRT-PCR and Western blotting after 48 hours of transfection.

### 2.5. Western Blot Analysis and Nuclear Protein and Fractionation

Each sample was prepared using a lysis buffer containing 50 mM Tris-HCl (Sigma-Aldrich), 150 mM NaCl (Sigma-Aldrich), 1 mM ethylenediaminetetraacetic acid (EDTA, Sigma-Aldrich), 1 mM egtazic acid (EGTA, Sigma-Aldrich), 0.1% sodium dodecyl sulfate (SDS, Sigma-Aldrich), 0.5% sodium deoxycholate (Sigma-Aldrich), 1% Triton X-100 (Sigma-Aldrich), and protease inhibitor cocktail (Sigma-Aldrich). Nuclear protein and fractionation were separated by cytoplasmic and nuclear protein extraction kit (BioTools Co. Ltd. Taiwan) according to manufacturer's instructions. Subsequently, the samples were separated on 12% SDS-polyacrylamide gels and were transferred onto polyvinylidene fluoride (PVDF) membranes (Millipore). After blocking, the membranes were incubated with the following primary antibodies: caspase 3 (1 : 500, Cell Signaling), NRF2 (1 : 1 000, Santa Cruz), NQO1 (1 : 1 000, Santa Cruz), GAPDH (1 : 10000, Proteintech), and H3 (1 : 2000, Cell Signaling) overnight at 4°C. Subsequently, the immune complexes were detected using relevant horseradish peroxidase-conjugated secondary antibodies (goat anti-mouse or goat anti-rabbit IgG antibody at 1 : 5 000, Santa Cruz) and an enhanced chemiluminescent substrate (ECL, BioTools Co. Ltd, Taiwan). GAPDH and H3 were used as the housekeeping loading control to total or nuclear protein assessment, respectively.

### 2.6. Immunofluorescent Staining and Measurement of Neurite Outgrowth

The cells cultured on coverslips were washed with phosphate-buffered saline (PBS, Invitrogen) and fixed with 4% paraformaldehyde (Sigma-Aldrich) for 10 minutes at room temperature. After three rinses (10 minutes each) with PBS containing Tween 20 (Invitrogen) (PBST), the cells were incubated in blocking solution consisting of PBST and 10% BSA (Sigma-Aldrich) for 30 minutes at room temperature. Subsequently, the samples were hybridized with the primary anti-TH (1 : 500, Millipore) and TUBB3 (1 : 500, Biolegend) antibodies in blocking solution overnight at 4°C. After three rinses (10 minutes each) with PBST, the cells were incubated with the diluted secondary antibody conjugated with Alexa 594 or Alexa 647 (Thermo Fisher Scientific) in blocking solution in the dark for 1 hour. The cells were counterstained with 4′-6-diamidino-2-phenylindole (DAPI, 1 : 1 000; Thermo Fisher Scientific) for nuclear detection. Subsequently, the coverslips were mounted with DAKO mounting solution onto microscopic slides. The cells were observed using a Leica TCS confocal microscope. The neurite outgrowth features (TH-positive) including total outgrowth, processes, and branches were counted randomly with more than 500 cells and assessed by MetaMorph microscopy automation and using the image analysis software (Molecular Devices).

### 2.7. Trypan Blue Cell Viability Assay

The cell viability was determined by light microscopy. Cells that were not stained with trypan blue were considered viable.

### 2.8. 3-[4,5-Dimethylthiazol-2-yl]-2,5-diphenyltetrazolium Bromide (MTT) Assay

The cells were incubated in 20 *μ*l MTT (Sigma-Aldrich) at 37°C for 2 hours. The absorbance of the purple formazan dye was detected at 570 nm using a microplate spectrophotometer.

### 2.9. LDH Assay

On day 14 after neuronal differentiation, cells were cultured with 100 *μ*l of LDH reaction mixture based on the manufacturer's instructions (Roche) and incubated at room temperature for 20 minutes. The samples' absorbance was set at 490 nm, and reading was performed by using a microplate spectrophotometer.

### 2.10. Caspase 3 Activity Assay

The Caspase 3 Assay Kit (Sigma-Aldrich) was used to measure the cellular caspase 3 activity based on the manufacturer's instructions. First, the cells were lysed in a lysis buffer composed of 50 mM 4-(2-hydroxyethyl)-1-piperazineethanesulfonic acid (HEPES, pH 7.4) (Sigma-Aldrich), 5 mM 3-[(3-cholamidopropyl)dimethylammonio]-1-propanesulfonate detergent (CHAPS; Sigma-Aldrich), and 5 mM dithiothreitol (DTT; Sigma-Aldrich) on ice for 20 minutes. After centrifugation, the supernatants containing proteins were quantified to evaluate the caspase 3 activity using Ac-DEVD-AMC as a fluorogenic substrate. The fluorescence wavelength of the AMC reading was set at 360 nm excitation in conjunction with a 460 nm emission filter.

### 2.11. Assessment of ROS

The cells were incubated at 37°C for 60 minutes in the Fluorogenic CellROX™ Deep Green Reagent (5 *μ*M, Molecular Probes), which is designed to measure ROS reliably in live cells. Subsequently, the cells were washed with PBS and the ROS levels were evaluated by measuring green fluorescence using a Leica TCS confocal microscope, with excitation/emission wavelengths of 488/520 nm. The cells were costained with TH, and only TH-positive cells were counted and analyzed.

### 2.12. Statistical Analyses

Data were presented as the means ± SD from three different passages and analyzed using Student's *t*-test or one-way analysis of variance (ANOVA) with Bonferroni's *post hoc* test. All statistical analyses were performed using the SPSS statistical software (version 18.0, IBM). Hierarchical clustering analysis was performed using the free academic software Cluster 3.0 (http://bonsai.hgc.jp/~mdehoon/software/cluster/software.htm#ctv). Significant statistical differences were considered at *p* < 0.05.

## 3. Results

### 3.1. Derivative NC001-8 Demonstrates No Neurotoxicity in DAergic Neurons

**Figure 1:**
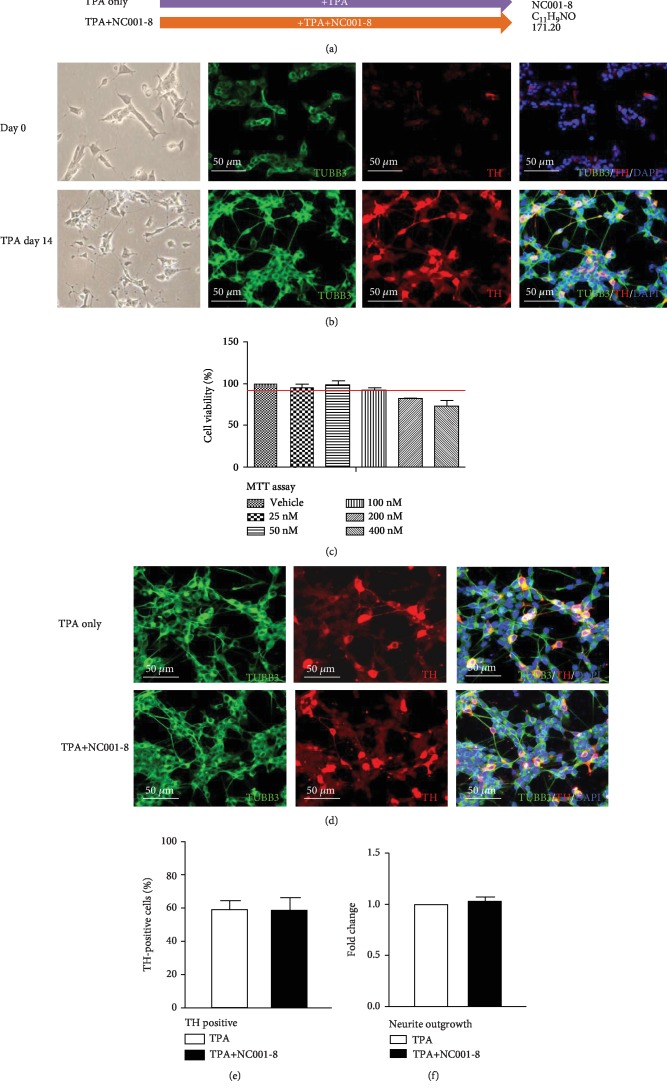
DAergic differentiation of SH-SY5Y cells. (a) Experiment flow chart of the differentiation of DAergic neurons from SH-SY5Y cells. Cells were incubated in the differentiation medium containing TPA (120 nM) and NC001-8 (100 nM) for 14 days. (b) Representative fluorescence microscopy images of differentiated DAergic neurons after 14 days of induction. Neurons expressed TUBB3 (green) and TH (red). (c) Drug cytotoxicity was estimated by using MTT assay, and the concentration of NC001-8 needed to maintain 90% of the growth was determined to be 100 nM. (d) Differentiated cells under NC001-8 treatment expressed TUBB3 and TH on day 14. (e) The percentage of TH-positive cells and (f) quantification of neurite outgrowth of DAergic neurons under NC001-8 treatment. Data were normalized to the treatment with TPA only and were represented as the means ± SD of three independent experiments. DAergic: dopaminergic; MTT: 3-[4,5-dimethylthiazol-2-yl]-2,5-diphenyltetrazolium bromide; TH: tyrosine hydroxylase; TPA: 12-O-tetradecanoyl-phorbol-13-acetate; TUBB3: tubulin beta 3 class III.

We applied 12-O-tetradecanoyl-phorbol-13-acetate (TPA) to induce the differentiation of SH-SY5Y cells into DAergic neurons [26]. The SH-SY5Y cells were cultured in a medium containing 120 nM TPA for 2 weeks (Figure 1(a)). On day 14, the cells had obviously changed morphologically and developed long neuritic processes. They also expressed the neuronal marker TUBB3 and the DAergic neuronal marker tyrosine hydroxylase (TH) (Figure 1(b)).

As an indole-derivative compound with an aromatic structure (Figure 1(a)), NC001-8, at a concentration of 100 nM, has demonstrated its neuroprotective potential in SCA cell models [22, 23]. Before examining the neuroprotective potential of NC001-8 in PD, we first evaluated its neurotoxicity in DAergic neurons. As shown in Figure 1(c), NC001-8 (concentration needed to be less cytotoxic for maintaining 90% of growth: 100 nM) was added to the medium during DAergic differentiation. After 14 days of differentiation, cells treated with NC001-8 also displayed the properties of DAergic neurons, including profound outgrowth of neurites and expression of TH and TUBB3 (Figure 1(d)). Treatment with NC001-8 did not affect the proportion of TH-positive cells (Figure 1(e)) and the level of neurite outgrowth (Figure 1(f)). These results suggested that NC001-8, at 100 nM, is not toxic to neurons and did not affect DAergic differentiation.

### 3.2. Treatment with NC001-8 Diminishes the ROS Level and Exhibits Potential for Neuroprotection in MPP^+^-Treated DAergic Neurons

**Figure 2:**
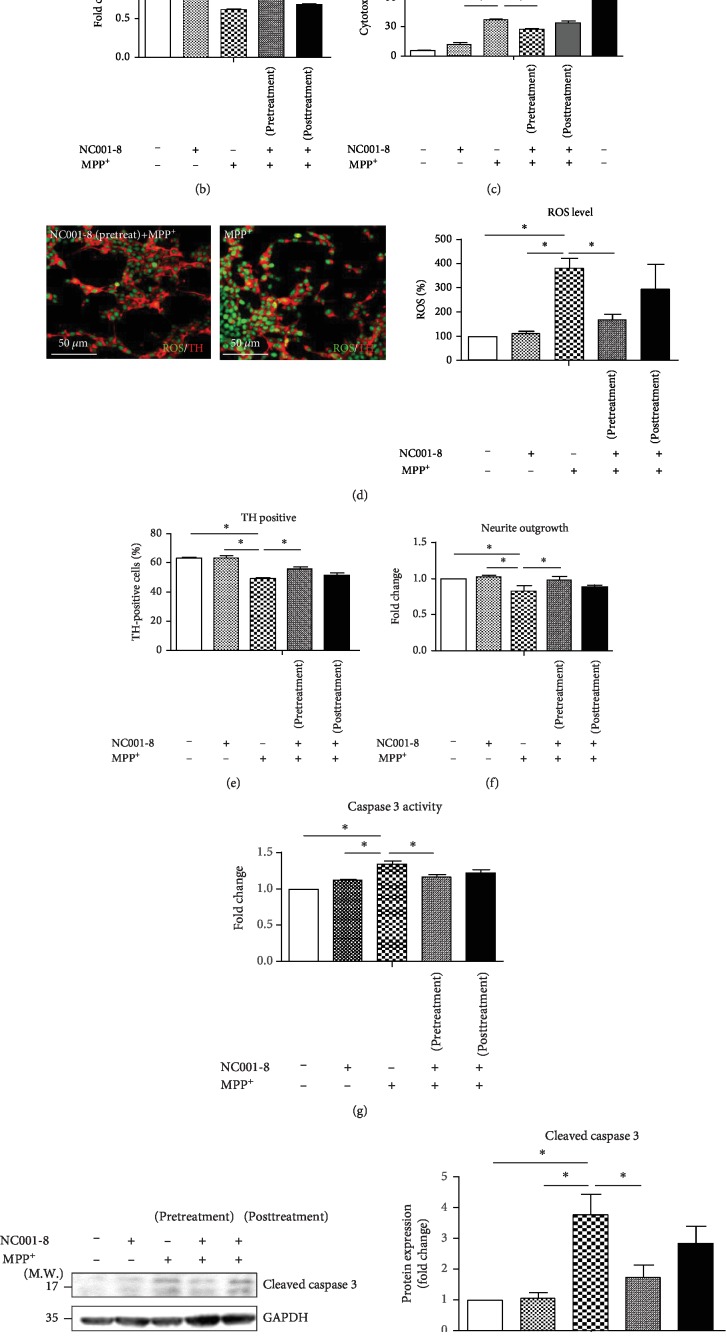
Neuroprotective effect of NC001-8 in SH-SY5Y-differentiated DAergic neurons. (a) Scheme of the experimental design. In the pretreatment group, cells were pretreated with NC001-8 during DAergic neuronal differentiation. On day 14, MPP^+^ (1 mM) was applied to induce death of DAergic neurons. In the posttreatment group, MPP^+^ was added to the medium between day 14 and day 16, followed by two-day treatment with NC001-8 (100 nM). (b) Cell viability (total counts with 2 × 10^4^ cells), (c) LDH assay (total counts with 2 × 10^4^ cells), (d) ROS production, (e) percentage of TH-positive cells (total counts with greater than 500 cells), (f) neurite outgrowth (total counts with greater than 500 cells), (g) caspase 3 activity assay, and (h) Western blot analysis of cleaved caspase 3 of DAergic neurons treated with MPP^+^ and/or NC001-8. Data were normalized to GAPDH and compared to cells with no treatment (*n* = 3, independent assays). ^∗^*p* < 0.05 (means ± SD). DAergic: dopaminergic; GAPDH: glyceraldehyde 3-phosphate dehydrogenase; LDH: lactate dehydrogenase; MPP^+^: 1-methyl-4-phenylpyridinium; ROS: reactive oxygen species; TH: tyrosine hydroxylase.

Previous reports have indicated that MPP^**+**^ selectively induces DAergic neuronal death and is widely used to establish PD cell models [27–29]. Here, MPP^+^ (1 mM) was added to SH-SY5Y-derived DAergic neurons from day 14 to day 16, accompanied by NC001-8 pre- or posttreatment (Figure 2(a)). Treatment with MPP^+^ significantly impaired cell viability (fold change: 0.62, *p* < 0.05 in comparison with no treatment, Figure 2(b)) and increased the levels of lactate dehydrogenase (LDH: 37%, *p* < 0.05 in comparison with no treatment; to normalize, whole cell lysis was set as 100%, Figure 2(c)). Pretreatment with NC001-8 reversed the reduction of cell viability (fold change: 0.78, *p* < 0.05 in comparison with MPP^+^ only, Figure 2(b)) and increased the level of LDH (26%, *p* < 0.05, in comparison with MPP^+^ only, Figure 2(c)). Compared with no treatment, treatment with MPP^+^ led to higher ROS levels (ROS production: 377%, *p* < 0.05, Figure 2(d)), reduced the proportion of TH-positive neurons (MPP^+^: 49%; no treatment: 63%, *p* < 0.05, Figure 2(e)) and neurite outgrowth (fold change: 0.83, *p* < 0.05, Figure 2(f)), and increased caspase 3 activity (fold change: 1.39, *p* < 0.05, Figure 2(g)) and the expression levels of cleaved caspase 3 (fold change: 3.77, *p* < 0.05, Figure 2(h)). Pretreatment with NC001-8 remarkably reduced the ROS levels (ROS production: 168%, *p* < 0.05 in comparison with MPP^+^ only, Figure 2(d)), increased the proportion of TH-positive neurons (56%, *p* < 0.05 in comparison with MPP^+^ only, Figure 2(e)) and neurite outgrowth (fold change: 0.97, *p* < 0.05 in comparison with MPP^+^ only, Figure 2(f)), and reduced caspase 3 activity (fold change: 1.08, *p* < 0.05 in comparison with MPP^+^ only, Figure 2(g)) and the expression level of cleaved caspase 3 (fold change: 1.73, *p* < 0.05 in comparison with MPP^+^ only, Figure 2(h)). On the other hand, posttreatment with NC001-8 did not induce any changes in cell viability, LDH and ROS levels, proportion of TH-positive neurons, neurite outgrowth, caspase activity, and expression levels of cleaved caspase 3. Therefore, we only applied NC001-8 pretreatment in the subsequent studies.

### 3.3. Pretreatment with NC001-8 Potentiates the NRF2 Antioxidative Pathway

**Figure 3:**
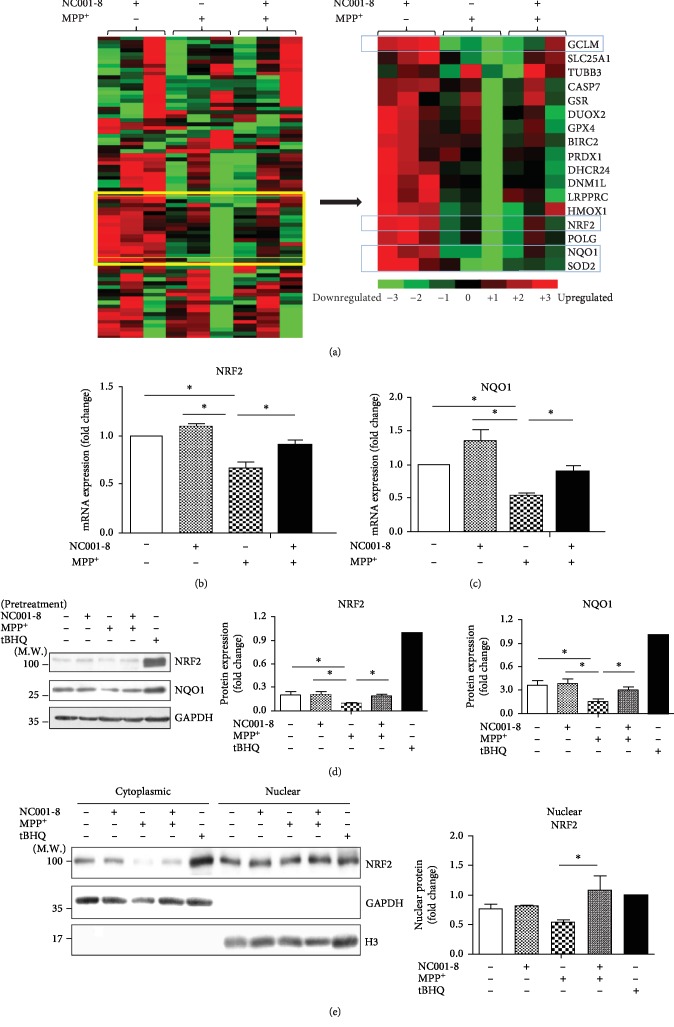
Enhancement of the NRF2 antioxidative pathway by NC001-8 in SH-SY5Y-differentiated DAergic neurons. (a) Heat map was representative for ROS-associated genes in 3 groups analyzed by clustering 3.0 software. Data were normalized to cells with no treatment. (b, c) Analysis of qRT-PCR and (d) Western blot of NRF2 and NQO1 in DAergic neurons treated with MPP^+^ and/or NC001-8/or tBHQ (25 *μ*M for 6 hours, as a positive control for NRF2 induction [41]). Data were normalized to GAPDH and compared to cells with no treatment (*n* = 3, independent assays). (e) Nucleus translocation of NRF2 in DAergic neurons treated with MPP^+^ and/or NC001-8/or tBHQ (25 *μ*M for 6 hours). Data were normalized to H3 and compared to cells with no treatment (*n* = 3, independent assays). ^∗^*p* < 0.05. tBHQ: tert-butylhydroquinone; qRT-PCR: quantitative reverse transcription-polymerase chain reaction; DAergic: dopaminergic; GAPDH: glyceraldehyde 3-phosphate dehydrogenase; MPP^+^: 1-methyl-4-phenylpyridinium; NRF2: nuclear factor erythroid 2-related factor 2; NQO1: NAD(P)H dehydrogenase, quinone 1.

To identify further key molecular targets of NC001-8 in ROS reduction and neuroprotection, we examined the expression alterations using an in-house quantitative polymerase chain reaction (q-PCR) array that carried 83 candidate genes involved in antioxidative and chaperon pathways (supplementary table) using SH-SY5Y-derived DAergic neurons treated with MPP^+^ and/or NC001-8 (Figure 3(a)). By this array, we found four genes (*NRF2*, *NQO1*, *GCLM* (glutamate-cysteine ligase modifier subunit), and *SOD2* (superoxide dismutase 2)) significantly upregulated by pretreatment with NC001-8 in MPP^+^-treated neurons (*p* < 0.05, supplementary table). The validation by repeated q-PCR showed that treatment with MPP^+^ reduced the expression levels of *NRF2* (fold change, 0.70; *p* < 0.05 in comparison with no treatment; Figure 3(b)) and *NQO1* (fold change, 0.57, *p* < 0.05 in comparison with no treatment; Figure 3(c)), whereas pretreatment with NC001-8 reversed these effects (*NRF2*: fold change, 0.88; *p* < 0.05 in comparison with MPP^+^ only; *NQO1*: fold change, 0.90; *p* < 0.05 in comparison with MPP^+^ only; Figures 3(b) and 3(c)). On the other hand, the validation result did not demonstrate significant upregulation of *GCLM* and *SOD2* by pretreatment with NC001-8 in MPP^+^-treated neurons (data not shown). Western blot analysis further confirmed the reduction of NRF2 (fold change, 0.46; *p* < 0.05 in comparison with no treatment; Figure 3(d)) and NQO1 (fold change, 0.42; *p* < 0.05 in comparison with no treatment; Figure 3(d)) by MPP^+^ treatment, while pretreatment with NC001-8 also mitigated these effects (NRF2: fold change, 0.94; *p* < 0.05 in comparison with MPP^+^ only; NQO1: fold change, 0.83; *p* < 0.05 in comparison with MPP^+^ only; Figure 3(d)). Pretreatment with NC001-8 further improved nuclear translocation (nuclear NRF2: fold change, 1.47; *p* < 0.05 in comparison with MPP^+^ only; Figure 3(e)). These results indicated that NC001-8 demonstrate an antioxidative effect by enhancement of the NRF2 pathway through translocation of NRF2 into the nucleus. Although the q-PCR array showed the upregulation of *NRF2* and *NQO1* in the MPP^+^-untreated/NC001-8-treated neurons, the validation results did not demonstrate significant upregulations of NRF2 and NQO1 by pretreatment with NC001-8 in MPP^+^-untreated neurons (Figures 3(c)–3(e)). The dose and time-response experiments also showed that pretreatment with NC001-8 did not alter the expression of NRF2 and NQO1 in MPP^+^-untreated neurons (supplementary figure).

### 3.4. Knockdown of NRF2 Attenuates the Neuroprotective and Antioxidative Effects of NC001-8

**Figure 4:**
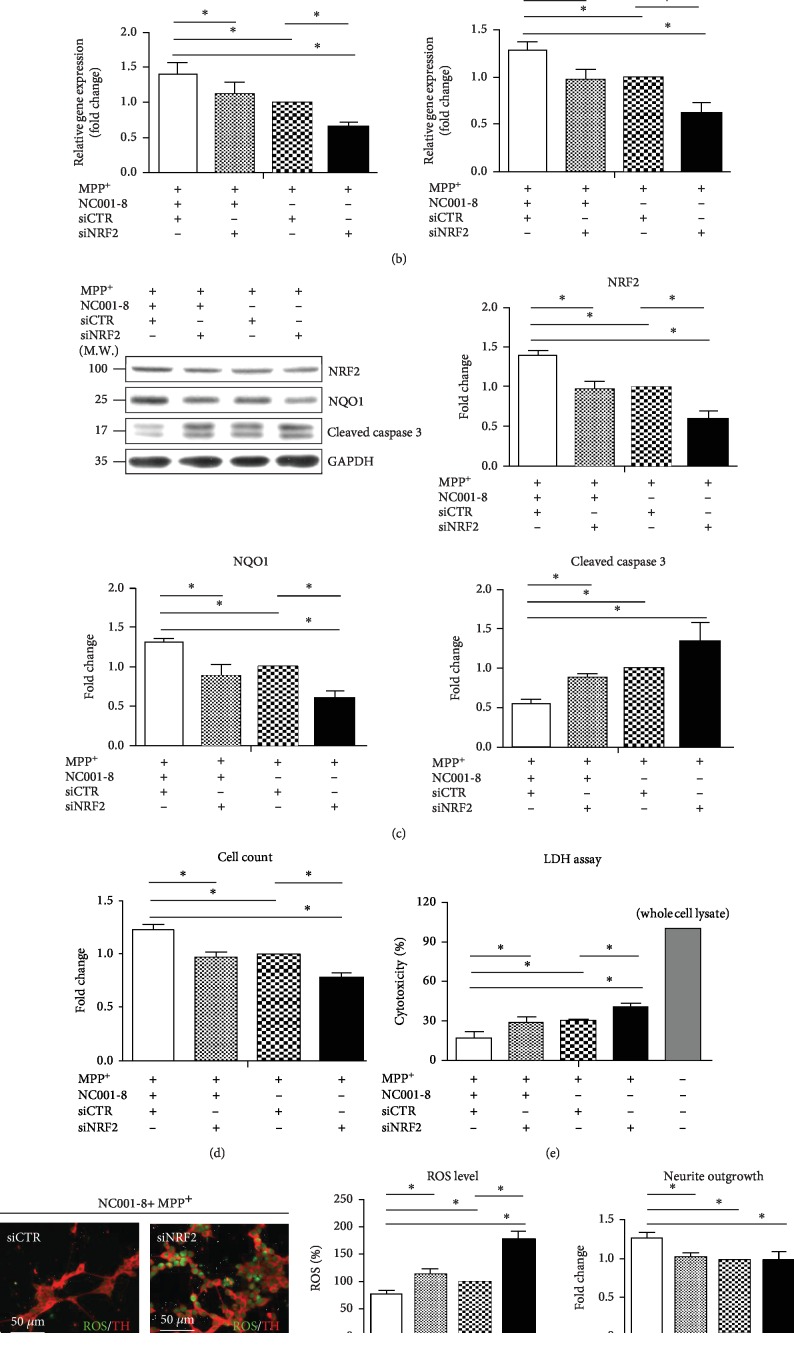
Counteraction of the neuroprotective and antioxidant effects of NC001-8 by knockdown of NRF2. (a) Scheme of the experimental design. The SH-SY5Y cells were treated with small interfering (si)RNA for *NRF2* from day 14 to day 16 followed by 2 days of 1 mM MPP^+^ treatment. (b) Analysis of qRT-PCR and (c) Western blot of NRF2, NQO1, and cleaved caspase 3 in DAergic neurons treated with MPP^+^, NC001-8, and/or *siNRF2*. Data were normalized to GAPDH and compared to cells with no treatment (*n* = 3, independent assays). (d) Cell viability (total counts with 2 × 10^4^ cells), (e) LDH assay (total counts with 2 × 10^4^ cells), (f) ROS level, and quantification of neurite outgrowth of DAergic neurons (total counts with more than 500 cells) treated with MPP^+^, NC001-8, and/or *siNRF2*. Data were normalized to MPP^+^-treated cells with scrambled control (siCTR) (*n* = 3, independent assays). ^∗^*p* < 0.05. DAergic: dopaminergic; GAPDH: glyceraldehyde 3-phosphate dehydrogenase; LDH, lactate dehydrogenase; MPP^+^: 1-methyl-4-phenylpyridinium; NRF2: nuclear factor erythroid 2-related factor 2; NQO1: NAD(P)H dehydrogenase, quinone 1; ROS: reactive oxygen species; qRT-PCR: quantitative reverse transcription-polymerase chain reaction.

To confirm the causality of neuroprotection and upregulation of the NRF2 pathway by NC001-8, we knockdown *NRF2* after NC001-8 administration in MPP^+^-treated DAergic neurons (Figure 4(a)). In MPP^+^-treated cells, knockdown of *NRF2* caused the reductions of *NRF2* (scrambled : knockdown, 1 : 0.68; *p* < 0.05; Figure 4(b)) and *NQO1* (scrambled : knockdown, 1 : 0.57; *p* < 0.05; Figure 4(b)). Following NC001-8, pretreatment significantly counteracted the upregulation of *NRF2* (scrambled : knockdown, 1.43 : 1.12; *p* < 0.05; Figure 4(b)) and *NQO1* (scrambled : knockdown, 1.32 : 0.97; *p* < 0.05; Figure 4(b)). Western blotting demonstrated consistent results in the protein levels (NRF2: scrambled : knockdown, 1.40 : 0.97; *p* < 0.05; and NQO1: scrambled : knockdown, 1.32 : 0.88; *p* < 0.05; Figure 4(c)). Knockdown of *NRF2* also counteracted the downregulation of cleaved caspase 3 (scrambled : knockdown, 0.55 : 0.89; *p* < 0.05; Figure 4(c)), increased cell viability (scrambled : knockdown, 1.21 : 0.98; *p* < 0.05; Figure 4(d)) and neurite outgrowth (scrambled : knockdown, 1.27 : 1.02; *p* < 0.05; Figure 4(f)), and reduced cytotoxicity (LDH: scrambled: 16%, knockdown: 30%, *p* < 0.05, Figure 4(e)) and ROS levels (scrambled: 78%, knockdown: 114%, *p* < 0.05, Figure 4(f)) by NC001-8 pretreatment. These findings consolidated the neuroprotective and antioxidative effects of NC001-8 via regulating the NRF2 pathway.

### 3.5. Treatment with NC001-8 Demonstrates Neuroprotective and Antioxidative Effects in the PD-iPSC Model

**Figure 5:**
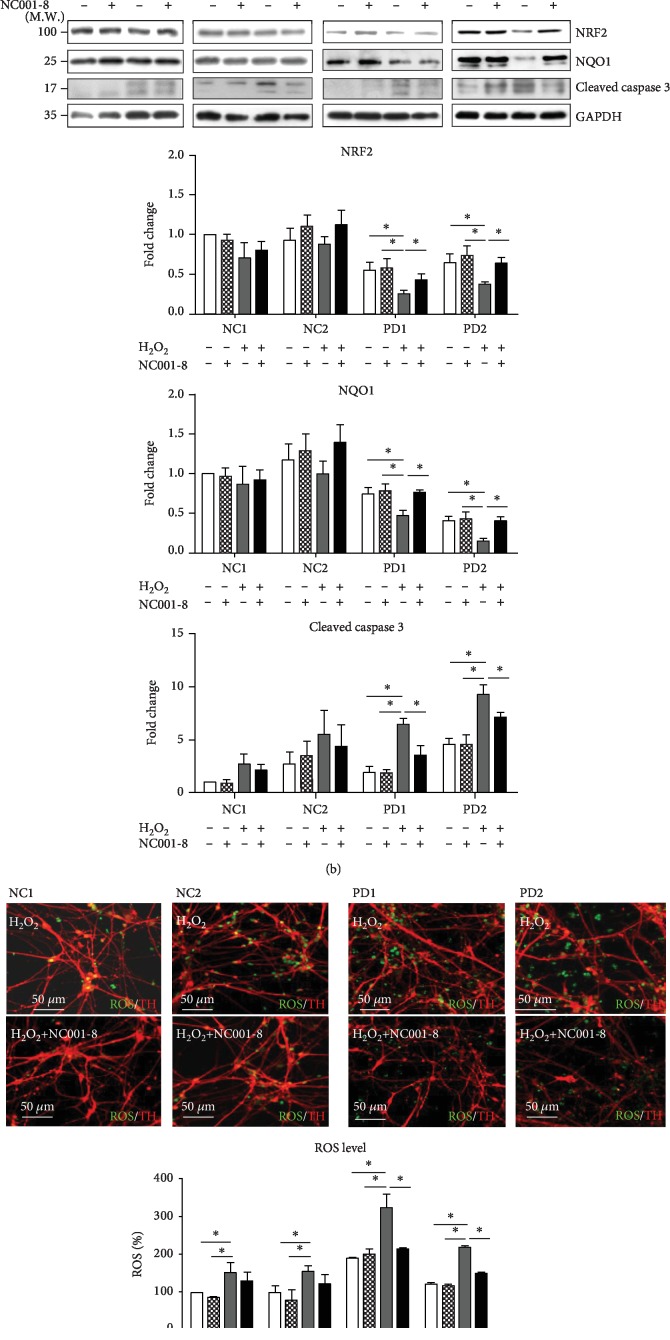
Neuroprotective and antioxidative effects of NC001-8 in PD-iPSC-derived DAergic neurons. (a) Scheme of the experimental design. DAergic neurons derived from PD-iPSCs were treated with NC001-8 (100 nM) before H_2_O_2_ (100 *μ*M) treatment for 8 hrs. (b) Western blot of NRF2, NQO1, and cleaved caspase 3 in DAergic neurons derived from NC- and PD-iPSCs treated with H_2_O_2_ and/or NC001-8. Data were normalized to GAPDH and compared to NC1 with no treatment. (c) The production of ROS in DAergic neurons treated with H_2_O_2_ and NC001-8. Data were normalized to NC1 with no treatment (over 500 cells were counted). Above these results were presented as the means ± SD from three different passages; ^∗^*p* < 0.05. NC-iPSC: induced pluripotent stem cells derived from a healthy volunteer; PD-iPSC: induced pluripotent stem cells carrying a *PARKIN* ex5del mutation; GAPDH: glyceraldehyde 3-phosphate dehydrogenase; NRF2: nuclear factor erythroid 2-related factor 2; NQO1: NAD(P)H dehydrogenase, quinone 1; ROS: reactive oxygen species.

We have previously generated PD-iPSCs carrying a *PARKIN* ex5del mutation [24]. The DAergic neurons derived from PD-iPSCs displayed abnormal susceptibility to H_2_O_2_ [24]. Here, we applied these PD-iPSC-derived DAergic neurons to validate the neuroprotective and antioxidative effects of NC001-8 (Figure 5(a)). Consistent with previous findings [24], treatment with H_2_O_2_ significantly reduced the expression levels of NRF2 (PD1: fold change, 0.24; PD2: fold change, 0.38; *p* < 0.05; Figure 5(b)) and NQO1 (PD1: fold change, 0.48; PD2: fold change, 0.13; *p* < 0.05; Figure 5(b)) and increased the levels of cleaved caspase 3 (PD1: fold change, 6.47; PD2: fold change, 9.32; *p* < 0.05; Figure 5(b)) when compared to untreatment in DAergic neurons derived from PD-iPSCs. Pretreatment with NC001-8 successfully improved the expression levels of NRF2 (PD1, fold change, NC001-8 : H_2_O_2_, 0.44 : 0.24; PD2, fold change, NC001-8 : H_2_O_2_, 0.64 : 0.38, *p* < 0.05 in comparison with no treatment of NC1, Figure 5(b)) and NQO1 (PD1, fold change, NC001-8 : H_2_O_2_, 0.76 : 0.48; PD2, fold change, NC001-8 : H_2_O_2_, 0.40 : 0.13; *p* < 0.05 in comparison with no treatment of NC1; Figure 5(b)) and reduced the upregulation of cleaved caspase 3 (PD1, fold change, NC001-8 : H_2_O_2_, 3.51 : 6.47; PD2, fold change, NC001-8 : H_2_O_2_, 7.09 : 9.32; *p* < 0.05 in comparison with no treatment of NC1; Figure 5(b)). Levels of ROS were significantly decreased by the treatment with NC001-8 in PD-iPSC-derived neurons (PD1, H_2_O_2_: 324%, NC001-8: 214%; PD2, H_2_O_2_: 219%, NC001-8: 150%; *p* < 0.05; Figure 5(c)). These findings consolidated the neuroprotective and antioxidative potentials of NC001-8 in DAergic neurons derived from PD patients.

## 4. Discussion

Currently, effective treatments that modify neurodegeneration in PD are still lacking. By using our DAergic neuronal models derived from SH-SY5Y cells and PD-iPSCs, we demonstrated for the first time the role of the indole derivative NC001-8 as an antioxidant to protect DAergic neurons. In SH-SY5Y-derived DAergic neurons, NC001-8 significantly attenuated the toxic effects of MPP^+^ by mitigating the ROS overproduction, neuronal apoptosis, and impairment of neurite outgrowth. Similar neuroprotective effects of NC001-8 were also observed in H_2_O_2_-treated DAergic neurons derived from PD-iPSCs. These results suggested therapeutic potentials of NC001-8 in PD.

To elaborate the mechanisms by which NC001-8 demonstrated its neuroprotective effect, we performed q-PCR analysis, which indicated that NC001-8 could improve the downregulation of *NRF2* and *NQO1* in SH-SY5Y-differentiated DAergic neurons under MPP^+^ treatment. Knockdown of *NRF2* in SH-SY5Y-differentiated DAergic neurons diminished the protective effects of NC001-8 against MPP^+^ toxicity, suggesting a regulation of these effects by the NRF2 antioxidative pathway. In H_2_O_2_-treated PD-iPSC-derived DAergic neurons, NC001-8 consistently decreased the levels of cleaved caspase 3, accompanied with increased expression levels of NRF2 and NQO1. These results further supported the notion that the upregulation of the NRF2 pathway by NC001-8 may protect DAergic neurons against environmental oxidative stress. Given that the expression of NRF2 is also downregulated in various models of Alzheimer's disease, SCA, and Parkinson's disease [30–35], further studies will be necessary to explore the neuroprotective potentials and molecular mechanisms of NC001-8 in other neurodegenerative diseases.

One of the critical challenges in the research on PD is the lack of live DAergic neurons from patients for mechanistic studies and new drug discovery. The iPSCs derived from patients with PD can recapitulate disease phenotypes to serve as a platform for testing new potential therapeutic strategies [24, 36–38]. The PD-iPSC-derived DAergic neurons demonstrated low expression levels of NRF2 and NQO1, in addition to a higher susceptibility to the environmental stressor such as H_2_O_2_, thus serving as a good model to evaluate the effects of NC001-8. Treatment with NC001-8 reduced the overproduction of ROS and expression of cleaved caspase 3 by H_2_O_2_ toxicity and upregulated NRF2 and NQO1 in PD-iPSC-derived DAergic neurons, suggesting its neuroprotective and antioxidative potentials in DAergic neurons from patients with PD. Similarly, we have previously demonstrated that genipin improved the abnormal susceptibility of PD-iPSC-derived DAergic neurons to H_2_O_2_ treatment by activating the NRF2 pathway [24]. Thus, strategies to induce the expression of antioxidative genes such as *NRF2* or *NQO1* could be a viable approach to develop neuroprotective therapies for PD.

Although we have shown that NC001-8 exerted neuroprotection via the enhancement of the NRF2 antioxidative pathway, pleiotropic effects of this compound may also lead to its neuroprotective effects. For example, NC001-8 also attenuated oxidative stress and polyQ-mediated neurotoxicity by upregulating chaperones including HSF1, HSPA1A, and HSP70 [22, 23]. However, our q-PCR array did not reveal these expression alterations in MPP^+^-treated DAergic neurons. The molecular mechanism to control NRF2 expression is still not well understood, although KRAS, BRAF, and MYC have been reported to upregulate *NRF2* expression via binding to the *NRF2* promoter site [39]. Future genome-wide expression studies are warranted to explore whether there is any other underlying mechanism contributing to the neuroprotective effects of NC001-8 in PD.

Our results also indicated that NC001-8 exerts its neuroprotective effects only when applied before MPP^+^ treatment in SH-SY5Y-derived DAergic neurons (pretreatment), suggesting a concept for an early window of therapeutic intervention in PD before exposure to environmental hazards. However, the clinical presentations of PD appear when approximately 50% of the DAergic neurons are lost [40]. Identification of diagnostic markers for preclinical PD is crucial to ensure effective intervention during a limited period early in the course of the disease for preventing subsequent disease progression.

In conclusion, our *in vitro* study provided evidence that the indole derivative NC001-8 could be a novel compound for PD treatment through the activation of the NRF2 antioxidative pathway. However, the NC001-8 neuroprotective effects and disease-modifying potentials should be further validated *in vivo* in PD animal models. The heterogeneous nature of PD may also limit the generalization of this neuroprotective strategy to all patients with PD. In the future, development of personalized medicine may be valuable for identifying possible patient candidates responsive to this therapeutic strategy.

## Data Availability

The data used to support the findings of this study are available from the corresponding author upon request.

## Supplementary Materials

Supplementary Materials Supplementary Table: the gene list of the q-PCR array for relevant ROS profiling. Supplementary Figure: dose and time response of NRF2 and NQO1 expression by treatment with NC001-8 in SH-SY5Y-differentiated DAergic neurons. Western blot of NRF2 and NQO1 in DAergic neurons treated with NC001-8 (25, 50, 100, and 200 *μ*M) for 7, 14, and 18 days. Data were normalized to GAPDH and compared to cells with no treatment (*n* = 3, independent assays).
