# Supplementary material for: Neuroprotection of Indole-Derivative Compound NC001-8 by the Regulation of the NRF2 Pathway in Parkinson's Disease Cell Models
Source: Oxid Med Cell Longev. 2019 Oct 31;2019:5074367. doi: 10.1155/2019/5074367 (PMC6874971; doi:10.1155/2019/5074367)
Supplement: Supplementary Materials — Supplementary Table: the gene list of the q-PCR array for relevant ROS profiling. Supplementary Figure: dose and time response of NRF2 and NQO1 expression by treatment with NC001-8 in SH-SY5Y-differentiated DAergic neurons. Western blot of NRF2 and NQO1 in DAergic neurons treated with NC001-8 (25, 50, 100, and 200 μM) for 7, 14, and 18 days. Data were normalized to GAPDH and compared to cells with no treatment (n = 3, independent assays). [file 5074367.f1.doc]

**Supplementary material for:**

**Neuroprotection of indole-derivative compound NC001-8 by regulation of NRF2 pathway in Parkinson's disease cell models**

Pei-Cih Wei^1^, Guey-Jen Lee-Chen^2^, Chiung-Mei Chen^1^, Yih-Ru Wu^1^, Yi-Jing Chen^1^, Jia-Li Lin^1^, Yen-Shi Lo^1^, Ching-Fa Yao^3^, *Kuo-Hsuan Chang^1^

^1^Department of Neurology, Chang Gung Memorial Hospital-Linkou Medical Center, Chang Gung University School of Medicine, Taoyuan, Taiwan

^2^Department of Life Science, National Taiwan Normal University, Taipei, Taiwan

^3^Department of Chemistry, National Taiwan Normal University, Taipei, Taiwan

**Correspondence:**

*Kuo-Hsuan Chang, MD., Ph.D.

Phone: +886-3-3281200 ext. 8421

Fax: +886-3-3288849

E-mail: [Gophy5128@cgmh.org.tw](mailto:knoxtn@cgmh.org.tw)

Supplementary Table ***Gene list of q-PCR array for relevant ROS profiling***

| **Gene symbol** | **Gene ID** |  |
| --- | --- | --- |
| AIFM2 | 84883 | apoptosis inducing factor, mitochondria associated 2 |
| AIP | 9049 | aryl hydrocarbon receptor interacting protein |
| ALDH2 | 217 | aldehyde dehydrogenase 2 family (mitochondrial) |
| APAF1 | 317 | apoptotic peptidase activating factor 1 |
| BAD | 572 | BCL2 associated agonist of cell death |
| BAK1 | 578 | BCL2 antagonist/killer 1 |
| BAX | 581 | BCL2-associated X protein |
| BCL10 | 8915 | B-cell CLL/lymphoma 10 |
| BCL2 | 596 | B-cell CLL/lymphoma 2 |
| BCL2A1 | 597 | BCL2 related protein A1 |
| BCL2L1 | 598 | BCL2 like 1 |
| BCL2L10 | 10017 | BCL2 like 10 |
| BCL2L11 | 10018 | BCL2 like 11 |
| BCL2L13 | 23786 | BCL2 like 13 |
| BCL2L2 | 599 | BCL2 like 2 |
| BID | 637 | BH3 interacting domain death agonist |
| BIRC2 | 329 | baculoviral IAP repeat containing 2 |
| BNIP3 | 664 | BCL2/adenovirus E1B 19kDa interacting protein 3 |
| BOK | 666 | BCL2-related ovarian killer |
| CASP2 | 835 | caspase 2 |
| CASP3 | 836 | caspase 3 |
| CASP5 | 838 | caspase 5 |
| CASP6 | 839 | caspase 6 |
| CASP7 | 840 | caspase 7 |
| CASP8 | 841 | caspase 8 |
| CASP8AP2 | 9994 | caspase 8 associated protein 2 |
| CASP9 | 842 | caspase 9 |
| CYGB | 114757 | cytoglobin |
| DHCR24 | 1718 | 24-dehydrocholesterol reductase |
| DNAJB1 | 3337 | DnaJ heat shock protein family (Hsp40) member B1 |
| DNM1L | 10059 | dynamin 1-like |
| DUOX2 | 50506 | dual oxidase 2 |
| FADD | 8772 | Fas associated via death domain |
| FAS | 355 | Fas cell surface death receptor |
| FASLG | 356 | Fas ligand |
| FOXM1 | 2305 | forkhead box M1 |
| **Gene symbol** | **Gene ID** |  |
| FTH1 | 2495 | ferritin, heavy polypeptide 1 |
| GCLC | 2729 | glutamate-cysteine ligase catalytic subunit |
| GCLM | 2730 | glutamate-cysteine ligase modifier subunit |
| GPX1 | 2876 | glutathione peroxidase 1 |
| GPX2 | 2877 | glutathione peroxidase 2 |
| GPX3 | 2878 | glutathione peroxidase 3 |
| GPX4 | 2879 | glutathione peroxidase 4 |
| GPX7 | 2882 | glutathione peroxidase 7 |
| GSR | 2936 | glutathione reductase |
| GSS | 2937 | glutathione synthetase |
| HMOX1 | 3162 | heme oxygenase 1 |
| HMOX2 | 3163 | heme oxygenase 2 |
| HSF1 | 3297 | heat shock transcription factor 1 |
| HSP90AA1 | 3320 | heat shock protein 90kDa alpha family class A member 1 |
| HSPA4 | 3308 | heat shock protein family A (Hsp70) member 4 |
| HSPA5 | 3309 | heat shock protein family A (Hsp70) member 5 |
| HSPA8 | 3312 | heat shock protein family A (Hsp70) member 8 |
| HSPB1 | 3315 | heat shock protein family B (small) member 1 |
| HSPD1 | 3329 | heat shock protein family D (Hsp60) member 1 |
| IKBKB | 3551 | inhibitor of kappa light polypeptide gene enhancer in B-cells, kinase beta |
| LRPPRC | 10128 | leucine rich pentatricopeptide repeat containing |
| MAP3K5 | 4217 | mitogen-activated protein kinase kinase kinase 5 |
| MCL1 | 4170 | myeloid cell leukemia 1 |
| MFN1 | 55669 | mitofusin 1 |
| MFN2 | 9927 | mitofusin 2 |
| NRF1 | 4899 | nuclear respiratory factor 1 |
| NRF2 | 4780 | nuclear factor, erythroid 2 like 2 |
| NQO1 | 1728 | NAD(P)H quinone dehydrogenase 1 |
| OPA1 | 4976 | OPA1, mitochondrial dynamin like GTPase |
| PMAIP1 | 5366 | phorbol-12-myristate-13-acetate-induced protein 1 |
| POLG | 5428 | polymerase (DNA) gamma, catalytic subunit |
| PPARGC1A | 10891 | PPARG coactivator 1 alpha |
| PRDX1 | 5052 | peroxiredoxin 1 |
| PRDX2 | 7001 | peroxiredoxin 2 |
| PRDX3 | 10935 | peroxiredoxin 3 |
| PRDX4 | 10549 | peroxiredoxin 4 |
| **Gene symbol** | **Gene ID** |  |
| PRDX5 | 25824 | peroxiredoxin 5 |
| PRDX6 | 9588 | peroxiredoxin 6 |
| SLC25A14 | 9016 | solute carrier family 25 member 14 |
| SOD1 | 6647 | superoxide dismutase 1, soluble |
| SOD2 | 6648 | superoxide dismutase 2, soluble |
| TFAM | 7019 | transcription factor A, mitochondrial |
| TH | 7054 | tyrosine hydroxylase |
| TUBB3 | 10381 | tubulin beta 3 class III |
| UCP1 | 7350 | uncoupling protein 1 (mitochondrial, proton carrier) |
| UCP2 | 7351 | uncoupling protein 2 (mitochondrial, proton carrier) |
| UCP3 | 7352 | uncoupling protein 3 (mitochondrial, proton carrier) |


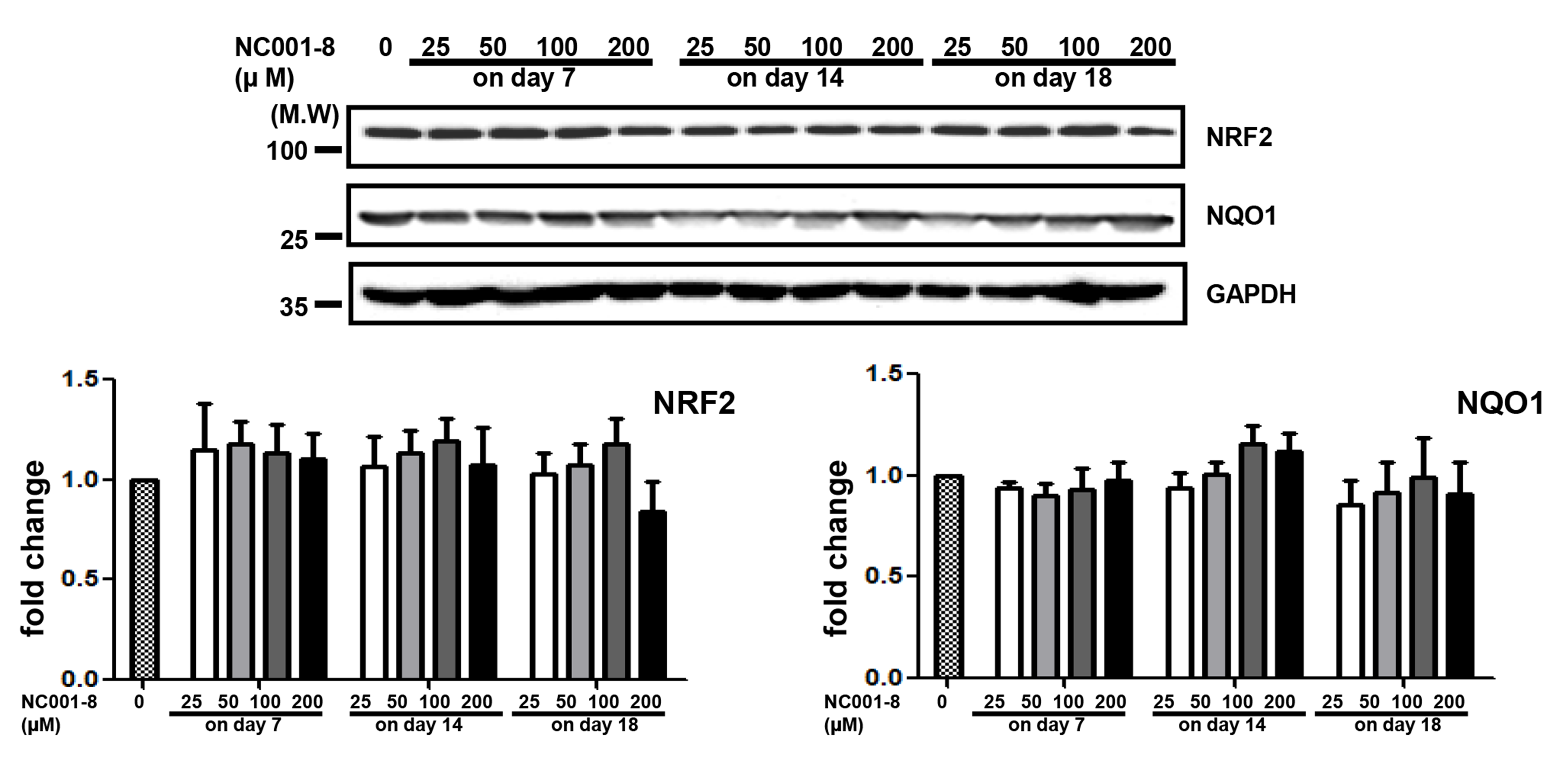


***Supplementary Figure. Dose and time-response of NRF2 and NQO1 expression by treatment with NC001-8 in SH-SY5Y-differentiated DAergic neurons.***

Western blot of NRF2 and NQO1 in DAergic neurons treated with NC001-8 (25, 50, 100, 200 μM) for 7, 14 and 18 days. Data were normalized to GAPDH and compared to cells with no treatment (n=3, independent assays).
